# Dieulafoy’s Lesion of the Ileum: Diagnostic Challenges and Surgical Management

**DOI:** 10.7759/cureus.111599

**Published:** 2026-06-27

**Authors:** Mouad Ouryemchi, Soufiane Taibi, Amal Bennani, Zahi Ismaili, Mohammed Bouziane

**Affiliations:** 1 Department of General Surgery, Mohammed VI University Hospital, Oujda, MAR; 2 Laboratory of Anatomy, Microsurgery and Surgery Experimental and Medical Simulation (LAMCESM), Faculty of Medicine and Pharmacy, Mohammed 1st University, Oujda, MAR; 3 Department of Pathology, Mohammed VI University Hospital, Oujda, MAR; 4 Department of Gastroenterology, Mohammed VI University Hospital, Oujda, MAR

**Keywords:** case report, dieulafoy ulcer, gastrointestinal hemorrhage, ileum, surgery

## Abstract

Dieulafoy's lesion is a rare cause of gastrointestinal hemorrhage characterized by submucosal arterial dilation without inflammatory changes. Ileal localization is exceptionally uncommon and poses significant diagnostic challenges. We report a case of a 50-year-old male patient who presented with upper gastrointestinal bleeding manifested by hematemesis and melena. Upper gastrointestinal endoscopy and colonoscopy failed to identify the bleeding source. Computed tomography angiography revealed a hyperdense nodular formation in the ileum. An exploratory laparotomy with bowel resection and end-to-end anastomosis was performed. Histopathological examination confirmed the diagnosis of Dieulafoy's lesion of the ileum. The patient was discharged on the third postoperative day with uneventful recovery and remained in good health at four-week follow-up. This case illustrates that an ileal Dieulafoy's lesion should be considered in the differential diagnosis of obscure gastrointestinal bleeding when endoscopic evaluation is inconclusive. Although enteroscopy or capsule endoscopy may aid in diagnosis, surgery remains the definitive diagnostic and therapeutic intervention in regions where these facilities are not available.

## Introduction

Dieulafoy's lesion is a rare and potentially life-threatening cause of gastrointestinal hemorrhage. Initially described by Gallard in 1884 as miliary aneurysms of the stomach, the lesion was later characterized by Georges Dieulafoy in 1898 as exulceratio simplex [1]. The pathophysiology remains incompletely understood, but the hallmark is submucosal fibrosis of dilated arteries with a diameter of 1 to 3 mm, notably devoid of inflammatory changes [2]. While gastric localization is most common, ileal involvement is exceedingly rare, representing less than 1% of cases [2]. This rarity renders diagnosis particularly challenging, as standard endoscopic evaluation often fails to detect the lesion. We present a case of an ileal Dieulafoy's lesion managed surgically after an unsuccessful endoscopic and imaging workup.

## Case presentation

A 50-year-old male patient with no significant medical history presented to the emergency department with upper gastrointestinal bleeding characterized by moderate hematemesis and melena. On examination, the patient was conscious and hemodynamically stable, with blood pressure of 110/70 millimeters of mercury and oxygen saturation of 92% on ambient air. Laboratory tests demonstrated microcytic hypochromic anemia with hemoglobin of 8.1 grams per deciliter. Other hematologic parameters were within normal limits (Table 1).

**Table 1:** Laboratory parameters

Parameter	Value	Reference Range	Unit
Hemoglobin	8.2	13.0 – 17.0	g/dL
Packed cell volume (PCV)	26	40 – 55	%
Mean corpuscular volume (MCV)	68	80 – 100	fL
Mean corpuscular hemoglobin concentration (MCHC)	28	32 – 36	g/dL
Platelets	234	150 – 400	×10³ cells /µL
White blood cell count (WBC)	17.6	4.0–10.0	×10³ cells /µL
Polymorphonuclear neutrophils (PMN)	9.7	1.5–7.0	×10³ cells/µL
C-reactive protein (CRP)	500	<5	mg/L
Procalcitonin (PCT)	0.27	<0.5	ng/mL
Serum creatinine (SCr)	0.9	0.6–1.2	mg/dL
Alanine aminotransferase (ALT)	18	7–56	U/L
Aspartate aminotransferase (AST)	17	10–40	U/L

**Figure 1:**
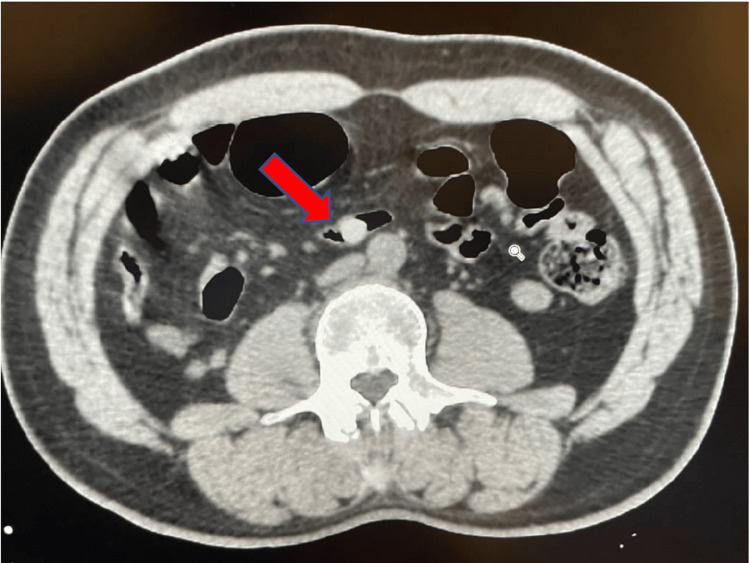
Computed tomography angiography showing hyperdense nodular formation in the ileum (arrow)

The patient underwent esophagogastroduodenoscopy, which revealed blood stasis in the stomach without active bleeding. Subsequent colonoscopy also failed to identify any bleeding source. Six hours later, follow-up testing revealed hemoglobin at 7 grams per deciliter. Computed tomography angiography was performed, uncovering a spontaneously hyperdense nodular formation in the ileum (Figure 1).

**Figure 2:**
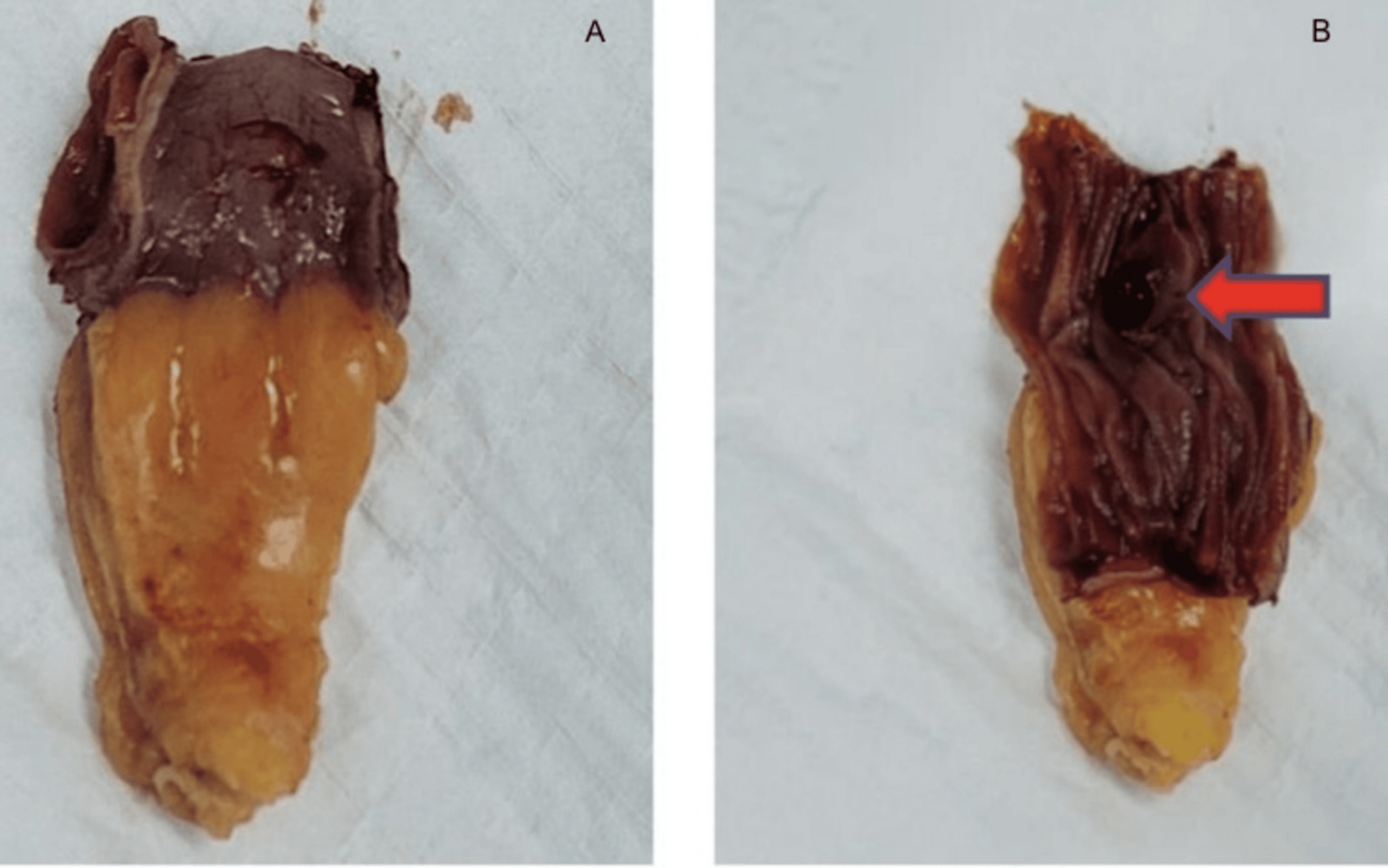
Resected bowel segment containing the nodular lesion (arrow) A: the macroscopic appearance of the resected ileal segment with the Dieulafoy lesion; B: the lesion after opening the specimen.

The case was discussed in a multidisciplinary meeting, and the decision was made to proceed with an exploratory laparotomy. The open approach was chosen to ensure optimal resection and reconstruction and minimize intraoperative risks, with concerns about underlying pathology necessitating direct visualization and tactile assessment. Intraoperative examination revealed a telangiectatic nodular formation located approximately two meters from the first jejunal loop (Figure 2). Bowel resection (7 cm), including the lesion (2.1 x 1.6 mm), with manual single-layer end-to-end entero-enteral anastomosis using 3/0 monofilament suture was performed.

Postoperative recovery was uneventful with no further bleeding. Follow-up hemoglobin after intraoperative transfusion was 10.2 grams per deciliter. The patient was discharged on the third postoperative day.

**Figure 3:**
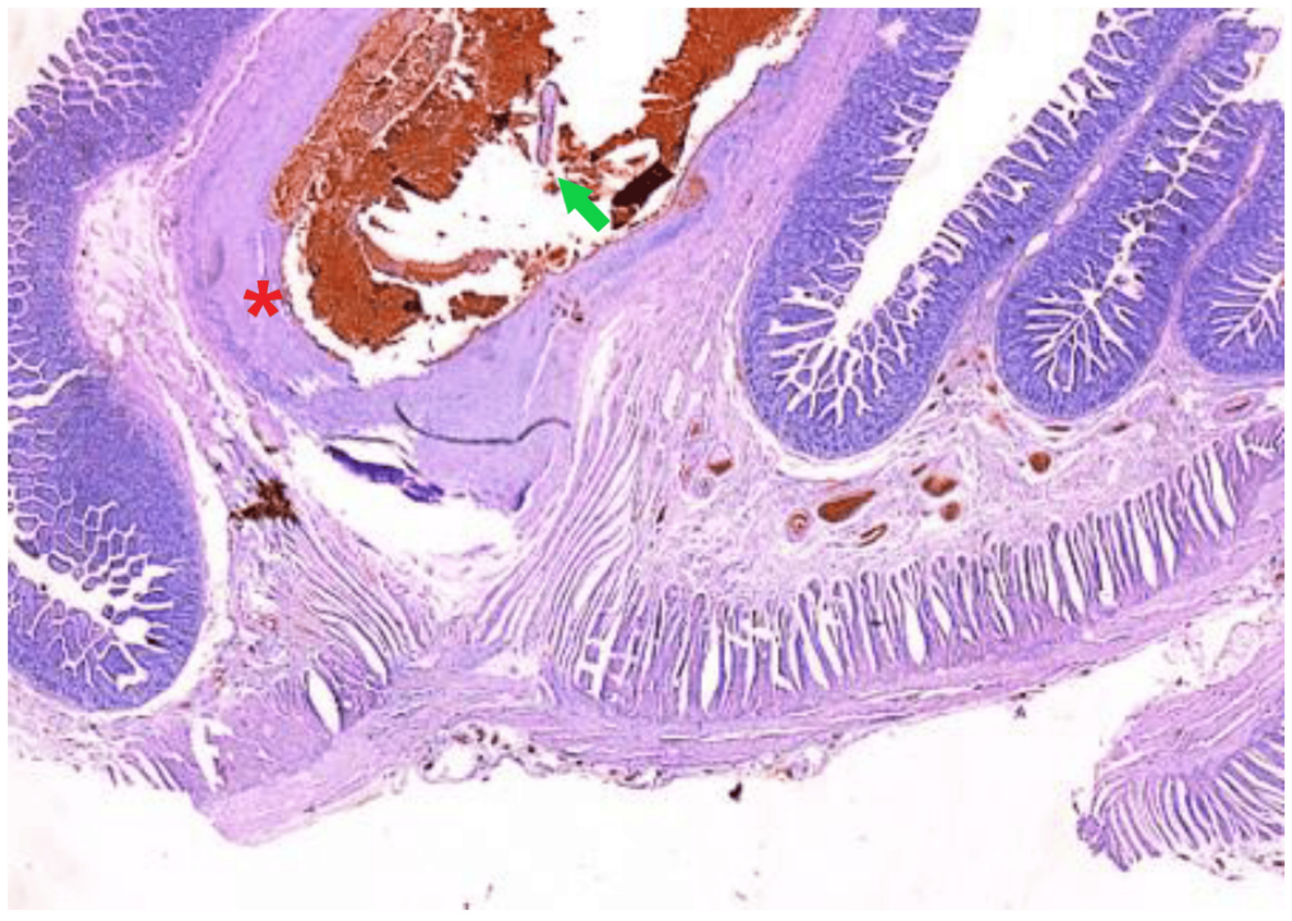
Histological section (hematoxylin-eosin staining), low magnification (x40) Shows a large, dilated arteriole in the submucosa (green arrow), lined by endothelial cells and featuring a thickened wall (*). H&E sections show an ectatic, large-caliber submucosal arteriole traversing the muscularis mucosae, with a superficial fibrin-platelet thrombus and minimal mucosal ulceration, without significant inflammation. These features confirm the diagnosis of Dieulafoy's lesion.

**Figure 4:**
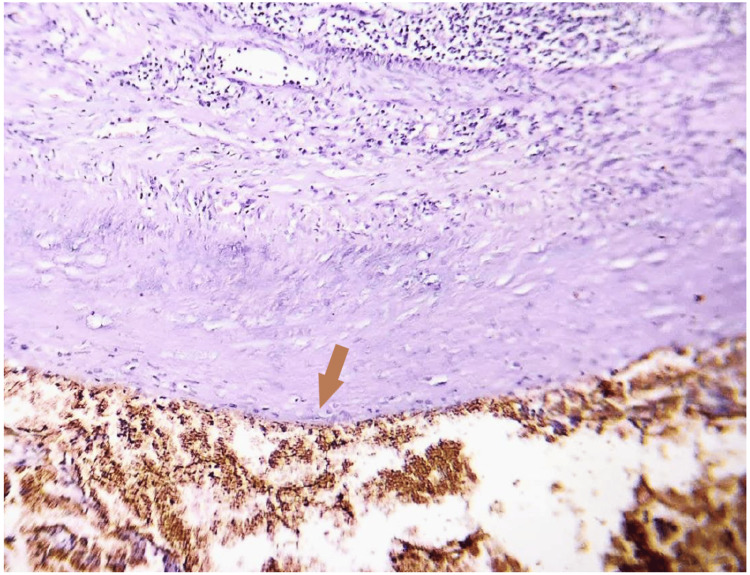
Histological section (hematoxylin-eosin staining), higher magnification (x100) Detailing the arterial wall (brown arrow) and the presence of endothelial cells.

Histopathological examination confirmed Dieulafoy's lesion with no sign of malignancy (Figures 3, 4). The patient presented for follow-up consultation one month postoperatively, reporting absence of melena or hematemesis. Clinical examination showed a clean surgical wound, stable vital signs, and good general condition. No further biochemical or imaging workup was indicated.

## Discussion

Dieulafoy's lesion, also known as a cirsoid aneurysm, accounts for 1% to 2% of gastrointestinal hemorrhage causes [2]. The most frequent location is the gastric, with other lesions described in the duodenum, jejunum, colon, and esophagus. Ileal localization remains very rare, with approximately 10 histologically proven cases reported in the literature [3]. There is no familial predisposition, and the condition is more common in elderly patients with cardiovascular comorbidities, hypertension, diabetes mellitus, and renal or hepatic failure. Males are affected twice as often as females [4].

Clinical presentation is variable and may range from asymptomatic incidental discovery during anemia workup to life-threatening massive gastrointestinal hemorrhage [5]. The standard diagnostic workup includes biological tests demonstrating microcytic hypochromic anemia, followed by endoscopic evaluation. Esophagogastroduodenoscopy and colonoscopy can be effective for gastric, duodenal, and colonic locations, typically revealing a large-diameter vascular stump (1 to 3 mm) protruding through a small mucosal erosion (2 to 5 mm), often covered with clots [2]. However, these modalities may be inconclusive when the lesion is very small or located in areas inaccessible to endoscopy, such as the ileum in our case.

In such instances, computed tomography angiography remains a reasonable diagnostic tool, capable of detecting active bleeding or a polypoid nodule, though no specific radiological signs exist [6]. Video capsule endoscopy is limited by availability. Technetium-99m-labeled red blood cell scans have been utilized to locate bleeding Dieulafoy's lesions when endoscopy is unsuccessful, offering the advantage of detecting extravasation at just 20% of the threshold required for angiography [7,8]. These tests were not used in our patient due to their unavailability at our center.

The role of angiography remains debated, given its diagnostic and therapeutic potential, but the failure rate is high, with recurrence rates of 60% to 70%, necessitating repeat endoscopy or surgical intervention [9]. Ultimately, the diagnosis is usually confirmed by histological study, which demonstrates abnormally dilated vessels in the submucosa and ruptured vessels in the intestinal wall [10].

Several endoscopic techniques have been employed, including sclerotherapy, epinephrine injection, thermocoagulation with bipolar and heating probes, argon plasma coagulation, and mechanical methods [11]. Surgery is currently reserved for approximately 5% of cases refractory to endoscopic methods or for inaccessible localizations. Surgical management consists of segmental resection with anastomosis, as in our case of ileal localization. Some authors suggest that surgical resection might be preferable in specific situations, particularly for lesions with a hemorrhagic tendency, although it is not systematically recommended as first-line therapy [12].

Our case does not allow generalization; the video capsule was unavailable, and although surgery was the gold standard, its role has diminished with endoscopy, but it remains indicated in emergencies for inaccessible or refractory lesions.

## Conclusions

Ileal Dieulafoy's lesion is rare and should be considered in the setting of obscure gastrointestinal hemorrhage. Endoscopy is currently the procedure of choice for diagnosis and treatment, but surgery retains an important role for lesions inaccessible to endoscopic evaluation. This case highlights the value of surgery as a diagnostic and therapeutic option when conventional modalities fail, without, however, making this a general rule.
